# RE: Intestinal Parasitic Infections in Human Immunodeficiency Virus-Infected and Noninfected Persons in a High Human Immunodeficiency Virus Prevalence Region of Cameroon

**DOI:** 10.4269/ajtmh.17-0859

**Published:** 2018-02

**Authors:** Shannan N. Rich

**Affiliations:** Department of Epidemiology; College of Public Health and Health Professions; College of Medicine; University of Florida; Gainesville, FL

Dear Sir,

I read with great interest the recent article entitled “Intestinal Parasitic Infections in Human Immunodeficiency Virus-Infected and Noninfected Persons in a High Human Immunodeficiency Virus Prevalence Region of Cameroon” published in September 2017. The authors examined the prevalence of and risk factors associated with intestinal parasitic coinfections among persons living with HIV (PLHIV) in the Adamaoua region of Cameroon.^1^ Previous studies conducted in various regions of sub-Saharan Africa have revealed disproportionately higher prevalence rates of parasitic infections among PLHIV.^2–4^ It is, therefore, surprising that the authors of this study reported no association between HIV status and parasitic infection. There are several factors that could have contributed to the null finding, which justifies further investigation.

First, given that the study participants were recruited in hospitals, consideration of the participants’ comorbid conditions is warranted. Of interest in this study is whether subjects were presenting with diarrhea at the time of stool collection and whether presence of this condition differed by HIV status. In addition, among PLHIV, the authors did not distinguish between prevalent and incident cases—increasing the risk of selection bias. The likelihood of parasitic coinfection in PLHIV varies by immune status; prevalence and density of parasites have been shown to increase according to CD4 T-cell declines.^2,5^ The authors reported an HIV incidence rate in their population nearly twice as high as in the target population.^1^ This suggests that newly infected PLHIV may have been overrepresented in this sample and their immunocompetence—relative to prevalent PLHIV—could have biased the observed association toward the null.

Second, the authors stated that study participants were not taking antiparasitic medications, but it is unclear whether their assessments included the antibiotic cotrimoxazole which has antiparasitic properties and is commonly prescribed to PLHIV to prevent opportunistic infections.^6^ Cotrimoxazole has demonstrated antiparasitic activity against multiple intestinal parasites including *Isospora belli*,^7^ which was tested for but not detected here.^1^ In a recent study conducted in Yaounde, Cameroon, cotrimoxazole prophylaxis was reported among 65.9% of PLHIV between 2015 and 2016.^6^ Cotrimoxazole use among PLHIV in this study may have decreased parasite prevalence in this group, thereby diluting the observed association.

Future studies investigating parasitic coinfection in PLHIV should consider the immune status and use of cotrimoxazole prophylaxis in this population, in addition to the presence of comorbidities in the overall population. The overall prevalence of intestinal parasites was 32.3% in this study,^1^ which suggests that routine screening and treatment of intestinal parasites could benefit the population as a whole.
